# Polypharmacy during pregnancy and associated risk factors: a retrospective analysis of 577 medication exposures among 1.5 million pregnancies in the UK, 2000-2019

**DOI:** 10.1186/s12916-022-02722-5

**Published:** 2023-01-16

**Authors:** Anuradhaa Subramanian, Amaya Azcoaga-Lorenzo, Astha Anand, Katherine Phillips, Siang Ing Lee, Neil Cockburn, Adeniyi Francis Fagbamigbe, Christine Damase-Michel, Christopher Yau, Colin McCowan, Dermot O’Reilly, Gillian Santorelli, Holly Hope, Jonathan I. Kennedy, Kathryn M. Abel, Kelly-Ann Eastwood, Louise Locock, Mairead Black, Maria Loane, Ngawai Moss, Rachel Plachcinski, Shakila Thangaratinam, Sinead Brophy, Utkarsh Agrawal, Zoe Vowles, Peter Brocklehurst, Helen Dolk, Catherine Nelson-Piercy, Krishnarajah Nirantharakumar, on behalf of the MuM-PreDiCT Group

**Affiliations:** 1 Institute of Applied Health Research, University of Birmingham, Birmingham, B15 2TT UK; 2 Division of Population and Behavioural Sciences, School of Medicine, University of St Andrews, St Andrews, UK; 3 Department of Epidemiology and Medical Statistics, College of Medicine, University of Ibadan, Ibadan, Nigeria; 4 Medical and Clinical Pharmacology, School of Medicine, Université Toulouse III, Toulouse, France; 5 INSERM, Center for Epidemiology and Research in Population Health (CERPOP), Toulouse, CIC 1436 France; 6 Division of Informatics, Imaging and Data Sciences, Faculty of Biology Medicine and Health, The University of Manchester, Manchester, UK; 7 Health Data Research UK, Oxford, UK; 8 Centre for Public Health, Queen’s University of Belfast, Belfast, UK; 9 Bradford Institute for Health Research, Bradford, UK; 10 Centre for Women’s Mental Health, Division of Psychology and Mental Health, School of Health Sciences, Faculty of Biology Medicine & Health, The University of Manchester, Manchester, UK; 11 Data Science, Medical School, Swansea University, Swansea, UK; 12 Greater Manchester Mental Health NHS Foundation Trust, Manchester, UK; 13 St Michael’s Hospital, University Hospitals Bristol NHS Foundation Trust, Bristol, UK; 14 Health Services Research Unit, School of Medicine, Medical Science and Nutrition, University of Aberdeen, Aberdeen, UK; 15 Aberdeen Centre for Women’s Health Research, School of Medicine, Medical Science and Nutrition, University of Aberdeen, Aberdeen, UK; 16 Centre for Maternal, Fetal and Infant Research, The Institute of Nursing and Health Research, Ulster University, Coleraine, UK; 17 Patient and Public Representative, London, UK; 18 WHO Collaborating Centre for Global Women’s Health, Institute of Metabolism and Systems Research, University of Birmingham, Birmingham, UK; 19 Department of Obstetrics and Gynaecology, Birmingham Women’s and Children’s NHS Foundation Trust, Birmingham, UK; 20 Guy’s and St. Thomas’ NHS Foundation Trust, London, UK

**Keywords:** Multiple medications, Polypharmacy, Medications, Prescriptions, Pregnancy, Multimorbidity, Multiple long-term conditions

## Abstract

**Background:**

The number of medications prescribed during pregnancy has increased over the past few decades. Few studies have described the prevalence of multiple medication use among pregnant women. This study aims to describe the overall prevalence over the last two decades among all pregnant women and those with multimorbidity and to identify risk factors for polypharmacy in pregnancy.

**Methods:**

A retrospective cohort study was conducted between 2000 and 2019 using the Clinical Practice Research Datalink (CPRD) pregnancy register.

Prescription records for 577 medication categories were obtained. Prevalence estimates for polypharmacy (ranging from 2+ to 11+ medications) were presented along with the medications commonly prescribed individually and in pairs during the first trimester and the entire pregnancy period.

Logistic regression models were performed to identify risk factors for polypharmacy.

**Results:**

During the first trimester (812,354 pregnancies), the prevalence of polypharmacy ranged from 24.6% (2+ medications) to 0.1% (11+ medications). During the entire pregnancy period (774,247 pregnancies), the prevalence ranged from 58.7 to 1.4%.

Broad-spectrum penicillin (6.6%), compound analgesics (4.5%) and treatment of candidiasis (4.3%) were commonly prescribed. Pairs of medication prescribed to manage different long-term conditions commonly included selective beta 2 agonists or selective serotonin re-uptake inhibitors (SSRIs).

Risk factors for being prescribed 2+ medications during the first trimester of pregnancy include being overweight or obese [aOR: 1.16 (1.14–1.18) and 1.55 (1.53–1.57)], belonging to an ethnic minority group [aOR: 2.40 (2.33–2.47), 1.71 (1.65–1.76), 1.41 (1.35–1.47) and 1.39 (1.30–1.49) among women from South Asian, Black, other and mixed ethnicities compared to white women] and smoking or previously smoking [aOR: 1.19 (1.18–1.20) and 1.05 (1.03–1.06)]. Higher and lower age, higher gravidity, increasing number of comorbidities and increasing level of deprivation were also associated with increased odds of polypharmacy.

**Conclusions:**

The prevalence of polypharmacy during pregnancy has increased over the past two decades and is particularly high in younger and older women; women with high BMI, smokers and ex-smokers; and women with multimorbidity, higher gravidity and higher levels of deprivation. Well-conducted pharmaco-epidemiological research is needed to understand the effects of multiple medication use on the developing foetus.

**Supplementary Information:**

The online version contains supplementary material available at 10.1186/s12916-022-02722-5.

## Background

A rise in the prevalence of long-term health conditions and an increase in the average maternal age in the United Kingdom (UK) mean that women will potentially be prescribed an increasing number of medications during pregnancy [1, 2]. It has been observed in other high-income countries that the prescription of multiple medications has been on a rise, but there has been no literature reporting on this over the last decade or within the UK [3, 4]. While a number of studies on antenatal medication exposures have focused on individual medications, very few studies have looked at multiple medications or rates of polypharmacy among pregnant women, with even fewer studies describing rates by trimester or capturing the common combinations prescribed together [5–7].

The term polypharmacy broadly refers to the use of multiple medications; however, no consensus on this definition exists within the literature. A systematic review on definitions of polypharmacy found that the definitions within the literature were variable in terms of (1) the number of medications prescribed together in a given duration, ranging from 2 to 11 or more medications; (2) consideration of treatment duration within the definition; (3) restriction to a specific prescription setting; and (4) consideration of prescribing appropriateness [8]. In addition, studies on polypharmacy lack consistency as to whether routine vitamin supplementation should be included [3, 9–11]. As a result, there is a wide range in the estimated prevalence of polypharmacy reported during pregnancy within the literature [11–18].

With the increasing number of prescribed medications, there is an increased risk of inappropriate or high-risk prescription [19], medication interaction and thereby adverse outcomes [20, 21]. Notably, the impact of polypharmacy specifically during the pregnancy period is not well understood due to the limited availability of comprehensive medication safety data during pregnancy.

Multimorbidity is broadly defined as the presence of two or more long-term health conditions, although similar to polypharmacy there is a lack of consensus on its definition and which diseases to include in the definition [22]. Multiple medications may be required to manage each morbidity individually, thereby making polypharmacy a particular consequence of multimorbidity. Although more common among older adults, multimorbidity is increasing in the younger population, including women of reproductive age [23, 24]. Despite this, there is a dearth of literature regarding pregnant women with multimorbidity and none exploring polypharmacy in this population.

In this study, we define polypharmacy through a range of definitions, from 2 to 11 or more different medications prescribed during pregnancy (either throughout pregnancy or restricted to the first trimester) within primary care. Duration of treatment, dose and concomitance of drug use were not considered within the definition of polypharmacy used in this study. The objectives of this study were (1) to examine the prevalence of polypharmacy (based on a range of definitions) during pregnancy over the last two decades, (2) to describe which medications and combinations of medications are most commonly prescribed during pregnancy and (3) to evaluate the factors associated with polypharmacy during pregnancy. In a subgroup analysis, this study aimed to examine the burden of polypharmacy among pregnant women with active multimorbidity.

## Methods

### Design and data source

A population-based retrospective cohort study of pregnancies was conducted between 2000 and 2019 using the Pregnancy Register within Clinical Practice Research Datalink (CPRD) GOLD.

CPRD GOLD is a UK primary care database with anonymized medical records of over 20 million patients from 973 general practices. CPRD constitutes about 7% of the UK population and is broadly comparable to the entire UK population in terms of age and sex [25]. CPRD has developed an algorithm to retrospectively create a pregnancy register that lists pregnancy episodes using 4000 pregnancy-related Read and entity codes recorded within primary care [26]. Read codes constitute a hierarchical clinical coding system to document patients’ symptoms, diagnoses and referrals [27]. The algorithm, in addition to estimating the start and end dates of pregnancy, also estimates the start and end of the first, second and third trimesters and pregnancy outcomes for each pregnancy episode. Prescriptions issued by general practitioners (GPs) are recorded using drug codes that equate to a unique product (described by the product name).

### Study population

CPRD practices were deemed eligible 12 months after reporting up-to-standard date, the date at which the practice data are deemed to be of research quality [28]. Women were included in the study if they (1) had an acceptable patient flag (indicating sufficient data quality) [28], (2) were registered to an eligible general practice for a minimum of 12 months (to allow sufficient time to record patients’ baseline morbidity and demographic data) and (3) were aged between 15 and 49 years. Pregnancy episodes recorded within the eligibility period of eligible women formed the source cohort for this study. This included information on pregnancy start and outcome for each unique pregnancy episode.

Each pregnancy was followed up from the start date of the first trimester until the earliest of the following end points: (1) pregnancy end date, (2) date the pregnant woman was transferred out of her GP practice, (3) death or (4) cessation of practice data contribution to CPRD.

For the primary analysis, pregnancies with (1) complete follow-up during the first trimester and (2) complete follow-up during the entire pregnancy period (all three trimesters) formed the denominator population for two cohorts. Uniform and complete follow-up across the denominator cohort was established to limit time-window bias, whereby pregnant women with complete follow-up would have increased opportunity for their prescription data to be captured compared to pregnant women with incomplete follow-up. The first trimester cohort was specifically considered as the risk of teratogenicity and fetotoxicity due to prescription drugs are higher during early pregnancy.

Pregnancies without complete follow-up of their first trimester will include those that resulted in pregnancy loss. To avoid selective exclusion of these pregnancies, a sensitivity analysis was performed in which pregnancies with or without complete follow-up were included, and prescriptions until the end of their pregnancy were captured. Women were considered lost to follow-up if they transferred out of their GP practice, died or the practice they were registered with stopped contributing to the CPRD database.

Subgroup analysis was performed on pregnancies of women affected by multimorbidity. For this study, multimorbidity was defined as having 2 or more long-term physical or mental health conditions at the start of pregnancy. For long-term conditions that are episodic in nature, such as eczema, we considered them to be actively present at the start of pregnancy if they were diagnosed or treated for the same in the previous 12 months. The list of 79 morbidities and their individual phenome definitions to identify multimorbidity status are described elsewhere [29].

### Medication categories

The British National Formulary (BNF) is a hierarchical formulary of medications prescribed in the UK [30, 31]. Nearly 350,000 medications and medical products are available in the CPRD database’s product dictionary. CPRD has classified these products into a list of 1668 categories, assigning each product an eight-digit code broadly representing the BNF chapter and section the product fits into. When a product was classified into more than one category, it was randomly assigned to a category to keep the medication categories mutually exclusive of their product components. Two GP researchers (AA and SIL) reviewed the category list to capture and exclude non-medicinal categories, referring to dressings and devices, medications used for acute care within secondary care settings and low-dose folic acid supplementation, which is advisable in all pregnancies. The remaining 577 BNF categories, from here on referred to as medication categories, were used in the analysis.

### Statistical analysis

For each eligible pregnancy, the number of medication categories prescribed (out of the 577 medication categories) during the first trimester was counted. Polypharmacy prevalence during the first trimester for the overall study period was calculated as the number of eligible pregnancies with *n* number of medication categories prescribed during the first trimester (ranging from 2 to 11 or more medications) divided by the total number of eligible pregnancies with complete follow-up during the first trimester. Prevalence was then estimated annually for eligible pregnancies that were conceived in each year from 2000 to 2019. Cochran-Armitage test for prevalence trend was then conducted.

To identify the top 10 commonly prescribed medications during the first trimester, the proportion of pregnancies with a prescription for each medication category during the first trimester was calculated. This was done by dividing the number of pregnancies with a prescription record of the medication during the first trimester by the total number of pregnancies with complete follow-up during the first trimester.

From 577 individual medication categories, 166,176 medication pairs were possible. To identify the top 10 commonly prescribed medication pairs during the first trimester, the proportion of pregnancies with a prescription for each of the 166,176 possible medication pairs during the first trimester was estimated. The top 10 most common medication pairs prescribed for the management of two different long-term conditions were identified since the risk of polypharmacy is higher due to fragmented and specialist care of patients with multimorbidity. For this, two GP researchers (AA and SIL) independently screened the medication pairs and listed those that were prescribed for the management of two different long-term conditions.

In a set of post hoc analyses, (1) the proportions of pregnancies with a prescription record for each possible individual and medication pairs were estimated during the preconception period (90 days prior to the start of pregnancy) and (2) a series of logistic regression models were run to assess the association between various risk factors and polypharmacy defined as 2 and 5 or more medications. The risk factors considered were maternal age, pre-gravid BMI, ethnicity, smoking status, gravidity, country of registered practice, number of pre-existing morbidities and socio-economic status.

All tabulations were repeated for pregnancy cohorts with complete follow-up throughout pregnancy, sub-cohort of pregnancies affected by multimorbidity and all eligible pregnancies, i.e. pregnancies with or without complete follow-up. Stata IC, version 16 (StataCorp LLC), was used to curate and analyse the data.

## Results

Out of the 3,202,461 pregnancies available in the CPRD pregnancy register, 1,521,317 were eligible after implementation of the inclusion and exclusion criteria. Of these, 812,354 (53.4%) and 774,247 (50.9%) pregnancies had complete follow-up through the first trimester and the entire pregnancy respectively (Fig. S1).

### Baseline characteristics

Table 1 presents the baseline characteristics of pregnancies included in the primary analysis who were followed up through their first trimester period (*n*=812,354) and a subset of these pregnancies with multimorbidity [139,471 (17.2%)]. For the primary cohort, the mean age and body mass index (BMI) recorded prior to conception were 28.7 years (SD 6.0) and 25.3 kg/m^2^ (SD 5.6) respectively. The pregnancies were predominantly of women from White ethnic background (46.3%), although 46.2% of the pregnancies had missing ethnicity data. Out of all pregnancies, 14.0% and 25.8% were of women who were ex-smokers and smokers prior to conception. Based on obstetric history, 30.0%, 27.4%, 18.9%, 10.9% and 12.8% of the pregnancies were the 1st, 2nd, 3rd, 4th and 5th or higher pregnancy respectively (this includes pregnancy losses).

**Table 1:** Baseline characteristics of pregnancies included in the primary analysis and pregnancies of women with multimorbidity included in the subgroup analysis, followed up through their first trimester

Variable	All pregnancies	Pregnancies of women with multimorbidity
**Number of pregnancies**	**(*n*=812,354)**	**(*n*=139,471)**
**Age, years,** ***n*** **(%)**		
**15–19**	68,741 (8.46)	6344 (4.55)
**20–24**	151,734 (18.68)	22,575 (16.19)
**25–29**	225,289 (27.73)	37,982 (27.23)
**30–34**	232,213 (28.59)	42,468 (30.45)
**35–39**	113,694 (14)	24,537 (17.59)
**40–44**	19,480 (2.4)	5080 (3.64)
**45–49**	1203 (0.15)	485 (0.35)
**BMI, kg/m**^**2**^**,** ***n*** **(%)**		
**Underweight (<18.5)**	28,295 (3.48)	5079 (3.64)
**Normal weight (18.5–24.9)**	358,371 (44.12)	58,746 (42.12)
**Overweight (25–29.9)**	164,972 (20.31)	31,156 (22.34)
**Obese (>30)**	114,889 (14.14)	28,986 (20.78)
**Missing**	145,827 (17.95)	15,504 (11.12)
**Ethnicity,** ***n*** **(%)**		
**White**	376,454 (46.3)	69,104 (49.6)
**Black**	21,343 (2.6)	4905 (3.5)
**South Asian**	22,597 (2.8)	3445 (2.5)
**Mixed race**	4300 (0.5)	677 (0.5)
**Other race**	12,745 (1.6)	2468 (1.8)
**Ethnicity data missing**	374,915 (46.2)	58,872 (42.2)
**Smoking status,** ***n*** **(%)**		
**Non-smoker**	440,868 (54.3)	68,644 (49.2)
**Ex-smoker**	113,620 (14.0)	23,663 (17.0)
**Smoker**	209,927 (25.8)	43,634 (31.3)
**Smoking status missing**	47,939 (5.9)	3530 (2.5)
**Gravidity,** ***n*** **(%)**		
**1**	243,736 (30.0)	34,083 (24.4)
**2**	222,309 (27.4)	34,763 (24.9)
**3**	153,286 (18.9)	27,182 (19.5)
**4**	88,844 (10.9)	17,826 (12.8)
**5+**	104,179 (12.8)	25,617 (18.4)
**Country,** ***n*** **(%)**		
**England**	522,802 (64.7)	87,511 (62.7)
**Northern Ireland**	42,278 (5.2)	8127 (5.8)
**Scotland**	145,258 (17.9)	25,336 (18.2)
**Wales**	102,016 (12.6)	18,497 (13.3)
**IMD**		
**1 (most deprived)**	219,238 (26.99)	38,260 (27.43)
**2**	169,268 (20.84)	27,983 (20.06)
**3**	153,936 (18.95)	26,904 (19.29)
**4**	133,756 (16.47)	22,659 (16.25)
**5 (least deprived)**	136,156 (16.76)	23,665 (16.97)
**Number of pre-existing comorbidities, mean (SD)**	1.35 (1.48)	3.49 (1.47)

*BMI* Body mass index, *IMD* Index of Multiple Deprivation

### Polypharmacy prevalence

**Fig. 1:**
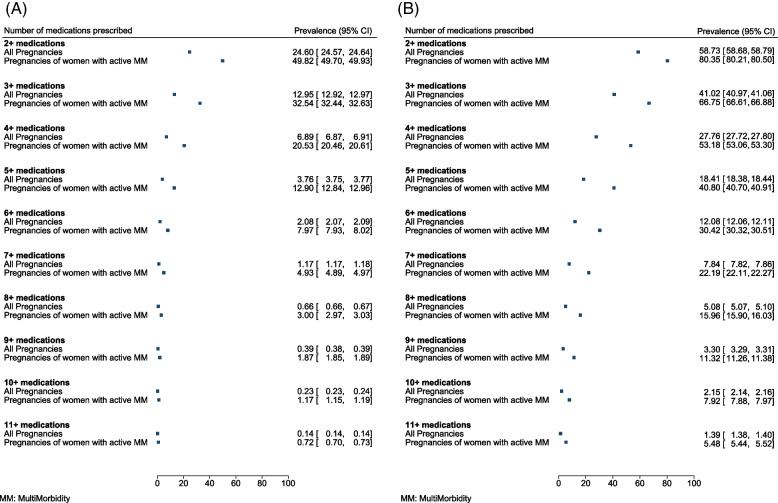
Polypharmacy prevalence according to a range of definitions (2 to 11 or more medications) during **A** the first trimester and **B** the entire pregnancy, among pregnancies of all eligible women and pregnancies of women with multimorbidity, who were followed up completely through the respective periods of prevalence estimation (primary analysis)

Figure 1 presents polypharmacy prevalence during the first trimester and the entire pregnancy for the overall study period (2000–2019), according to a pre-defined range of definitions. During the first trimester, 24.6% of all pregnancies [95% CI (24.6–24.6%)] and 49.8% (49.7–50.0%) of pregnancies of women with multimorbidity had two or more medications prescribed; 0.1% (0.1–0.1%) of all pregnancies and 0.7% (0.7–0.7%) of pregnancies of women with multimorbidity had 11 or more medications prescribed.

When considering the entire pregnancy period, 58.7% (58.7–58.8%) of all pregnancies and 80.3% (80.2–80.5%) of pregnancies of women with multimorbidity had two or more medications prescribed; 1.4% (1.4–1.4%) of all pregnancies and 5.5% (5.5–5.5%) of pregnancies of women with multimorbidity had 11 or more medications prescribed.

Figure S2 presents the polypharmacy prevalence from the sensitivity analyses including all pregnancies with or without complete follow-up.

### Annual polypharmacy prevalence trend

**Fig. 2:**
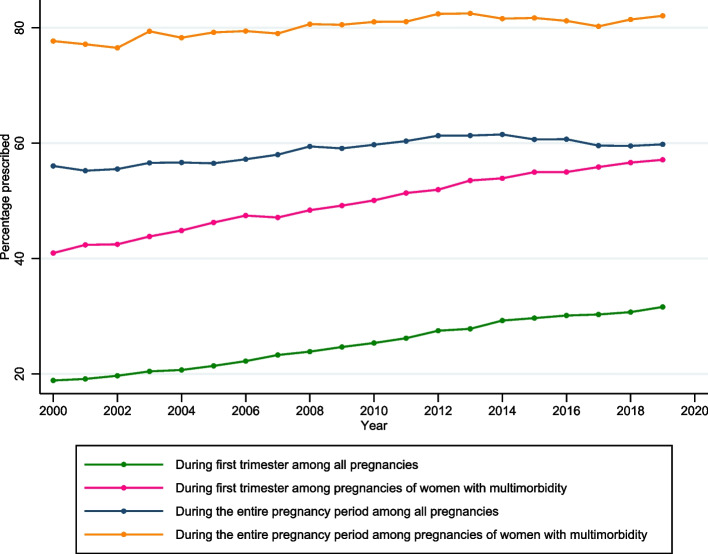
Polypharmacy (2 or more medications) prevalence trend from 2000 to 2019

Figure 2 presents the annual polypharmacy prevalence trend from 2000 to 2019 based on the polypharmacy definition of 2+ medications being prescribed. Among all pregnancies during the first trimester alone, the prevalence increased from 8.7 to 18.7% between 2000 and 2019. Among pregnancies of women with multimorbidity, during the first trimester, the prevalence increased from 24.3 to 39.8%. Although increments in polypharmacy were observed for the entire pregnancy period (*p*<0.001 from the Cochran-Armitage test), they were relatively less steep compared to the first trimester period. Figure S3 presents the annual polypharmacy prevalence trend based on the commonly used polypharmacy definition of 5+ medications being prescribed, and similar increasing trends are observed.

### Commonly prescribed medications

Table 2 presents the top 10 medication categories that were prescribed during the first trimester and the entire pregnancy among pregnant women with complete first trimester follow-up and the proportion of pregnancies prescribed with those medications. The proportion of all medications prescribed is available in Table S1.

**Table 2:** Top 10 medication categories that were commonly prescribed among all pregnancies and pregnancies of women with multimorbidity during first trimester and the entire pregnancy (primary analysis)

Period	All pregnancies		Pregnancies of women with multimorbidity	
	Medication categories	% of pregnancies prescribed	Medication categories	% of pregnancies prescribed
**First trimester**	**(*n*=812,354)**		**(*n*=139,471)**	
	Broad-spectrum penicillins	6.6%	Selective serotonin re-uptake inhibitors (SSRIs)	15.2%
	Non-opioid and compound analgesics	4.5%	Selective beta 2 agonists	11.3%
	Preparations for vaginal and vulval candidiasis	4.3%	Broad-spectrum penicillins	10.0%
	Selective serotonin re-uptake inhibitors (SSRIs)	4.1%	Non-opioid and compound analgesics	9.1%
	Selective beta 2 agonists	3.9%	Preparations for vaginal and vulval candidiasis	6.7%
	Topical corticosteroids	3.3%	Topical corticosteroids	6.3%
	Compound alginate preparations	3.3%	Thyroid hormones	5.9%
	Oral iron	3.2%	Compound alginate preparations	5.4%
	Products used against nausea and vertigo	2.8%	Corticosteroids (for respiratory conditions)	4.8%
	Cephalosporins	2.6%	Products used against nausea and vomiting	4.7%
**Entire pregnancy**	**(*n*=774,247)**		**(*n*=132,391)**	
	Oral iron	30.3%	Oral iron	32.5%
	Compound alginate preparations	19.1%	Broad-spectrum penicillins	26.0%
	Broad-spectrum penicillins	18.8%	Compound alginate preparations	24.7%
	Preparations for vaginal and vulval candidiasis	17.6%	Preparations for vaginal and vulval candidiasis	23.5%
	Non-opioid and compound analgesics	11.9%	Non-opioid and compound analgesics	19.7%
	Topical corticosteroids	8.4%	Selective beta 2 agonists	18.4%
	Cephalosporins	7.5%	Selective serotonin re-uptake inhibitors (SSRIs)	17.4%
	Selective beta 2 agonists	7.3%	Topical corticosteroids	13.7%
	Osmotic laxatives	7.0%	Osmotic laxatives	11.4%
	Selective serotonin re-uptake inhibitors (SSRIs)	4.9%	Cephalosporins	10.4%

NB: Women with complete follow-up throughout the first trimester and through the entire pregnancy were included in the respective cohorts. Medications are presented as CPRD-specific BNF medication categories

During the first trimester, the top three medications prescribed during pregnancy were broad-spectrum penicillins (6.6%), non-opioid compound analgesics (4.5%) and preparations for vaginal and vulval candidiasis (4.3%); when restricting to pregnancies of women with multimorbidity, the top three commonly prescribed medications were selective serotonin re-uptake inhibitors (SSRIs, 15.2%) and selective beta 2 agonists (11.3%) and broad-spectrum penicillins (10.0%). The common medications prescribed in the entire pregnancy were similar to the common medications prescribed in the first trimester.

In the sensitivity analysis presented in Table S2, the ten most commonly prescribed medications were broadly similar to those seen in the primary analysis. Progestogen products were prescribed in 4.7% of the pregnancies during trimester one.

### Commonly prescribed pairs of medications

Of the 166,176 possible pairs, a total of 29,470 pairs of medications were prescribed during the entire pregnancy. Table 3 presents the top 10 common medication pairs that were prescribed during the first trimester and the entire pregnancy among all pregnancies and pregnancies of women with multimorbidity. In addition, the medication pairs were screened to present those that are deemed to be prescribed for the management of two different long-term conditions.

**Table 3:** Top ten common medication pairs that were prescribed among all pregnancies and pregnancies of women with multimorbidity during first trimester and the entire pregnancy

Period	All pregnancies		Pregnancies of women with multimorbidity			
	Top 10 pairwise combinations prescribed		Top 10 pairwise combinations prescribed		Top 10 pairwise combinations prescribed for two different long-term conditions	
	Combinations of medication categories	% of pregnancies prescribed	Combinations of medication categories	% of pregnancies prescribed	Combinations of medication categories	% of pregnancies prescribed
**First trimester**	**(*n*=812,354)**		**(*n*=139,471)**			
	Corticosteroids (for respiratory conditions)	1.2%	Corticosteroids (for respiratory conditions)	3.4%	Selective serotonin re-uptake inhibitors (SSRIs)	1.8%
	Selective beta 2 agonists		Selective beta 2 agonists		Selective beta 2 agonists	
	Broad-spectrum penicillins	0.8%	Selective serotonin re-uptake inhibitors (SSRIs)	2.2%	Topical corticosteroids	1.4%
	Preparations for vaginal and vulval candidiasis		Non-opioid and compound analgesics		Selective beta 2 agonists	
	Broad-spectrum penicillins	0.8%	Broad-spectrum penicillins	2.1%	Corticosteroids used in nasal allergy	1.3%
	Non-opioid and compound analgesics		Selective beta 2 agonists		Selective beta 2 agonists	
	Broad-spectrum penicillins	0.7%	Selective serotonin re-uptake inhibitors (SSRIs)	1.8%	Non-sedating antihistamines	1.3%
	Selective beta 2 agonists		Selective beta 2 agonists		Selective beta 2 agonists	
	Emollient skin preparations	0.6%	Broad-spectrum penicillins	1.8%	Proton pump inhibitors	1.1%
	Topical corticosteroids		Selective serotonin re-uptake inhibitors (SSRIs)		Selective serotonin re-uptake inhibitors (SSRIs)	
	Non-opioid and compound analgesics	0.5%	Broad-spectrum penicillins	1.7%	Selective serotonin re-uptake inhibitors (SSRIs)	1.0%
	Non-steroidal anti-inflammatory drugs		Non-opioid and compound analgesics		Topical corticosteroids	
	Non-opioid and compound analgesics selective	0.5%	Non-opioid and compound analgesics	1.5%	Corticosteroids in chronic bowel problems	1.0%
	Serotonin re-uptake inhibitors (SSRIs)		Selective beta 2 agonists		Selective beta 2 agonists	
	Compound alginate preparations	0.5%	Broad-spectrum penicillins	1.5%	Selective serotonin re-uptake inhibitors (SSRIs)	0.8%
	Non-opioid and compound analgesics		Preparations for vaginal and vulval candidiasis		Thyroid hormones	
	Broad-spectrum penicillins	0.4%	Emollient skin preparations	1.4%	Non-sedating antihistamines	0.7%
	Selective serotonin re-uptake inhibitors (SSRIs)		Topical corticosteroids		Selective serotonin re-uptake inhibitors (SSRIs)	
	Non-opioid and compound analgesics	0.4%	Selective beta 2 agonists	1.4%	Emollient skin preparations	0.7%
	Preparations for vaginal and vulval candidiasis		Topical corticosteroids		Selective beta 2 agonists	
**Entire pregnancy**	**(*n*=774,247)**		**(*n*=132,391)**			
	Compound alginate preparations	7.2%	Compound alginate preparations	9.7%	Selective beta 2 agonists	3.7%
	Oral iron		Oral iron		Topical corticosteroids	
	Broad-spectrum penicillins	6.7%	Broad-spectrum penicillins	9.5%	Selective beta 2 agonists	3.2%
	Oral iron		Oral iron		Selective serotonin re-uptake inhibitors (SSRIs)	
	Oral iron	6.4%	Oral iron	8.9%	Corticosteroids used in nasal allergy	3.1%
	Preparations for vaginal and vulval candidiasis		Preparations for vaginal and vulval candidiasis		Selective beta 2 agonists	
	Broad-spectrum penicillins	5.2%	Broad-spectrum penicillins	8.5%	Selective serotonin re-uptake inhibitors (SSRIs)	2.3%
	Preparations for vaginal and vulval candidiasis		Preparations for vaginal and vulval candidiasis		Topical corticosteroids	
	Compound alginate preparations	4.7%	Broad-spectrum penicillins	7.8%	Corticosteroids in chronic bowel problems	2.3%
	Preparations for vaginal and vulval candidiasis		Compound alginate preparations		Selective beta 2 agonists	
	Broad-spectrum penicillins	4.7%	Non-opioid and compound analgesics	7.7%	Non-sedating antihistamines	2.2%
	Compound alginate preparations		Oral iron		Selective beta 2 agonists	
	Non-opioid and compound analgesics	4.6%	Compound alginate preparations	7.5%	Emollient skin preparations	2.1%
	Oral iron		Preparations for vaginal and vulval candidiasis		Selective beta 2 agonists	
	Broad-spectrum penicillins	3.9%	Broad-spectrum penicillins	7.4%	Proton pump inhibitors	1.8%
	Non-opioid and compound analgesics		Non-opioid and compound analgesics		Selective serotonin re-uptake inhibitors (SSRIs)	
	Compound alginate preparations	3.7%	Broad-spectrum penicillins	7.1%	Corticosteroids (for respiratory conditions)	1.7%
	Non-opioid and compound analgesics		Selective beta 2 agonists		Topical corticosteroids	
	Non-opioid and compound analgesics	3.2%	Compound alginate preparations	7.0%	Corticosteroids used in nasal allergy	1.6%
	Preparations for vaginal and vulval candidiasis		Non-opioid and compound analgesics		Topical corticosteroids	

NB: Women with complete follow-up throughout the first trimester and through the entire pregnancy were included in the respective cohorts. Medications are presented as CPRD-specific BNF medication categories. Combinations of medications that were considered to be prescribed for multimorbidity are medications that were used for the long-term physical or mental health conditions defined in our list of 79 conditions

When restricting to medications that were prescribed for the management of two different long-term conditions, SSRIs and selective beta 2 agonists (1.8%), topical corticosteroids and selective beta 2 agonists (1.4%) and corticosteroids used in nasal allergy and selective beta 2 agonists (1.3%) were commonly prescribed in the first trimester. All other medication pairs within the top 10 were either in combination with SSRIs or selective beta 2 agonists. Similar prescribing patterns for pairs of medications were observed for the entire pregnancy period.

In the sensitivity analysis, non-steroidal anti-inflammatory drugs (NSAIDs) and compound analgesics were prescribed in the first trimester in 0.54% of pregnancies. Otherwise, the common pairs identified in the sensitivity analysis were the same as those in the primary analysis. A list of all medications prescribed in pairs for the primary and sensitivity analyses is presented elsewhere in Table S3 and Table S4*.*

### Risk factors for polypharmacy

During the first trimester of pregnancy, a U-shaped relationship was observed between maternal age and odds of polypharmacy. Women aged 15–19 and 45–49 years were at significantly higher odds of being prescribed 2+ medications compared to those aged 30–34 years [aOR 2.16 (95% CI 2.12–2.21) and 1.86 (95% CI 1.64–2.10) respectively]. Compared to women with a record of normal pre-gravid BMI, women who were underweight, overweight and obese were at 8%, 16% and 55% higher odds of polypharmacy [aOR 1.08 (95% CI 1.05–1.11), 1.16 (95% CI 1.14–1.18 and 1.55 (95% CI 1.53-1.57) respectively]. Compared to women of white ethnicity, women of black, South Asian, mixed and other ethnicities were at significantly higher odds of polypharmacy [aOR 1.71 (95% CI 1.65–1.76), 2.40 (95% CI2.33–2.47), 1.39 (95% CI 1.30–1.49) and 1.41 (95% CI 1.35–1.47) respectively]. Compared to non-smokers, women who were current or ex-smokers were at significantly higher odds of polypharmacy [aOR 1.19 (95% CI 1.18–1.20) and 1.05 (95% CI 1.03–1.06) respectively]. Women with higher gravidity were at higher odds compared to those who were gravida 1 [gravida 2: aOR 1.02 (95% CI 1.00–1.03), gravida 3: aOR 1.08 (95% CI 1.07–1.10), gravida 4: aOR 1.13 (95% CI 1.11–1.15) and gravida 5+ aOR 1.26 (95% CI 1.24–1.29)]. Women living in Northern Ireland, Scotland and Wales were at higher odds of polypharmacy than those living in England [aOR 2.09 (95% CI 2.05–2.14), 1.58 (95% CI 1.56–1.60) and 1.40 (95% CI 1.38–1.42) respectively]. An increasing number of morbidities was associated with increasing odds of polypharmacy. Women with two pre-existing morbidities had more than twice the odds of being prescribed two or more medications compared to women with no morbidities [aOR 2.59 (95% CI 2.55–2.63)]. Women living in the most deprived areas were at higher odds than women living in the least deprived areas [OR 1.56 (95% CI 1.54–1.59)].

Similar trends were observed when considering (1) the most commonly used numerical definition of polypharmacy as the outcome (5 or more medications), (2) the entire pregnancy period as a window for prescription and (3) the number of medications prescribed as a numerical outcome (Figs. S4 and S5).

## Discussion

### Main findings

This study used primary care electronic health records to describe polypharmacy prevalence and common medications prescribed during pregnancy in the UK. During the first trimester, where exposure to teratogens is of highest concern, one in four pregnancies of all women (24.6%) and one in two pregnancies of women with multimorbidity (49.8%) were prescribed two or more medications. During the entire pregnancy period, the prevalence of two or more medications being prescribed was even higher (58.7% and 80.3% respectively). Over the last two decades between 2000 and 2019, a significant increasing trend in the prevalence of polypharmacy was observed both among all pregnancies and pregnancies of women with multimorbidity.

Commonly prescribed medications during pregnancy were medications typically used to manage (1) pregnancy-related symptoms or illnesses such as oral iron, analgesia, laxatives, antiemetics and antacids; (2) bacterial or fungal infections such as broad-spectrum penicillin and preparation for vaginal and vulva candidiasis; (3) common mental health conditions such as SSRI; and (4) asthma and atopic conditions such as selective beta 2 agonists and topical corticosteroids.

The 10 most common pairs of medications prescribed to manage two different long-term health conditions for pregnant women with multimorbidity in the first trimester all included pairings with either SSRI or selective beta 2 agonist.

Pregnant women of younger or older age groups (<25 and >35 years) with higher pre-gravid BMI, higher levels of socio-economic deprivation, smoking or history of smoking and increasing levels of multimorbidity were associated with higher odds of prescription of 2 and 5 or more medications

### Comparison with existing literature

The prevalence of polypharmacy during pregnancy observed in this study (defined as 2 or more medications prescribed) (58.7%) was comparable to the findings from a study in the Netherlands (62.4%) [32]. However, this was higher than the estimates reported in population-based studies from Denmark (42.7%) [33], Ireland (29.4%) [12], China (9.2%) [9] and the Netherlands (4.9%) [34]. Similarly, the prevalence of polypharmacy for other numerical definitions estimated in this study is higher than the estimates reported by a Danish study (41.0% versus 2.7%, for the definition 3+ medication) [11], a North American study (27.8% versus 4.9%, for the definition 4+ medication) [3] and a Finnish study (2.2% versus 0.2%, for the definition 10+ medication) [14].

Several reasons could be attributable to the higher prevalence of polypharmacy during pregnancy reported in this study. A variety of methods have been used in the literature to capture prescription data including pharmacy records [9, 33, 34], national registries [11, 14, 15] and self-reported medication use [6, 12, 35–37]. In this study, we captured medications that were prescribed based on primary care records, which may or may not have been dispensed or taken, whereas in the other studies whereby pharmacy records or surveys were used, medication consumption would have been captured more accurately. Some of the studies collected data from an earlier time period than this study [11, 14], which corresponds with the lower prevalence of polypharmacy observed in the earlier period of our study, with an increasing trend thereafter. In our study, we included vitamins and minerals (apart from folic acid), which are commonly taken during pregnancy, although prescription for these recorded within primary care is likely to reflect therapeutic use (such as therapeutic dose of oral iron for iron deficiency) as opposed to supplements, which are generally purchased over the counter. These prescriptions were excluded from a number of other studies [3, 11, 38]. The wide range of prevalence estimates reported in the literature may also be attributable to the differences in practices and healthcare systems internationally, such as payment for prescriptions out of pocket deterring patients from requesting prescriptions and the types of drugs that are available over the counter.

Common medications and the prevalence of their prescriptions observed during pregnancy in our study are broadly comparable to other previous studies. This includes antibiotics and treatments for asthma, allergy and anaemia [13, 32, 35]. Some differences in our findings from previously published literature [6], such as lower prevalence of products used against nausea and vertigo, may reflect the purchase of these products over the counter as opposed to procurement through prescription, which are best captured through self-reports and surveys than through primary care records as in our study.

Some of the risk factors for polypharmacy during pregnancy observed in our study have previously been described in other studies by Zhang et al. [9] and Cleary et al. [12], including higher maternal age and smoking as risk factors for higher number of medications used and prescription of US Food and Drug Administration (FDA) category D/X medications (with a positive evidence of human foetal risk) during pregnancy. However, neither of the studies suggested younger maternal age as a risk factor.

### Strengths and limitations

This study has important strengths, including the large cohort size of 1.5 million eligible pregnancies and 812,354 of them with complete follow-up of their first trimester, from a primary care database that is broadly generalizable to the UK. To our knowledge, this is the first study reporting on polypharmacy prevalence and common prescriptions, both individually and in pairs during pregnancy (1) over the last two decades, (2) within the UK and (3) in a sub-cohort of pregnant women with multimorbidity.

However, our specified definition of polypharmacy (prescription of multiple medications, ranging from 2 to 11 or more medications within a pre-defined pregnancy period, either the first trimester or the entire pregnancy period) had its limitations. It was not possible to determine the appropriateness of the medications prescribed as the indications for medications are not available in our dataset and the size of the cohort would prohibit a case-by-case examination. We are also unable to determine whether medications were prescribed concomitantly. Furthermore, our definition of polypharmacy was based only on primary care prescriptions, we do not know if the medication was actually dispensed and taken [39] and we did not capture over the counter and secondary care medication that could have over- or under-estimated our findings respectively.

Of the pregnancies recorded in the CPRD GOLD pregnancy register, 52.4% were excluded based on standard practice exclusion criteria due to patient or practice ineligibility at the start of pregnancy. Furthermore, 22.1% of pregnancies were excluded due to the incomplete follow-up of their first trimester. To examine for potential selection bias, we conducted a sensitivity analysis to include all eligible pregnancies with or without complete follow-up, in which where we observed similar findings.

### Clinical and research implication

Polypharmacy is known to be associated with multimorbidity in the general population [40, 41]. However, much research on polypharmacy has focused on older people [42, 43], with less attention given to pregnant women and women of childbearing age. As observed in our analysis, pregnancy is often associated with the prescription of medications to manage common pregnancy-related symptoms and illnesses such as pain, nausea and dyspepsia. The need for these medications will further add to the medication burden for women with multimorbidity who may already be taking regular medications for their underlying long-term health conditions. This was confirmed in our study findings: the prevalence of polypharmacy was considerably higher among pregnancies of women with multimorbidity compared to all pregnancies in general and the relative difference in their prevalence increased with the number of medications considered in the polypharmacy definition (Fig. 1).

In women with multimorbidity, SSRI was the most prescribed medication during the first trimester (15.2%) and the seventh most common medication prescribed during the entire pregnancy period (17.4%). Also, among the common pairs of medication, SSRI was frequently observed. This reflects the high prevalence of mental health conditions among pregnant women with multimorbidity observed in our previous study [1]. Given the uncertainties of antidepressant treatment safety during pregnancy [44], and the competing risks of untreated mental health conditions [45], this area warrants further research.

Our findings assessing the risk factors for polypharmacy suggested women between the age of 25 and 34 were at the lowest risk of polypharmacy during pregnancy, with risk increasing with both increasing and decreasing maternal age away from the central pregnancy age of 25–34 years. Women who were pregnant during their teenage years had more than twice the odds of being prescribed multiple medications compared to women who were pregnant at the age of 30–34. This may be attributable to women below the age of 20 being entitled to free prescriptions [46]. Furthermore, teenage pregnant women are more likely to receive supplementation for prevention of iron deficiency anaemia and treatments for sexually transmitted infections [47].

There is growing recognition that medications for long-term conditions should be continued during pregnancy if it is safe and if the benefit outweighs the risk. Notably, the assessment of teratogenic risk is primarily focused on the use of individual medications. Less is known about the combined effect of medications taken concomitantly during pregnancy. To empower women and clinicians, we need more research on the effect of combined medications taken during pregnancy to the women and the foetus.

## Conclusions

The prevalence of polypharmacy has increased over the past two decades, particularly in women with multimorbidity. Risk factors for polypharmacy include younger or older maternal age away from the central pregnancy age of 25–34 years, overweight or obese BMI, smoking or history of smoking, higher gravidity, pre-existing morbidities and low socio-economic status. Well-conducted pharmaco-epidemiological research is needed to understand the combined effects of multiple medications on both maternal and foetal outcomes.

## Supplementary Information

**Additional file 1: Figure S1.** Flow diagram showing the selection of eligible pregnancies from the CPRD pregnancy register.

**Additional file 2: Figure S2.** Polypharmacy prevalence according to a range of definitions (2 to 11 or more medications) during (A) first trimester and (B) the entire pregnancy, among pregnancies of all eligible women and pregnancies of women with multimorbidity, with or without complete follow up (Sensitivity Analysis).

**Additional file 3: Figure S3.** Polypharmacy (5 or more medications) prevalence trend from 2000 to 2019.

**Additional file 4: Table S1.** Proportions of pregnancies with a prescribed medication – primary analysis.

**Additional file 5: Table S2.** Proportions of pregnancies with a prescribed medication – sensitivity analysis.

**Additional file 6: Table S3.** Proportions of pregnancies with prescribed medication pairs – primary analysis.

**Additional file 7: Table S4.** Proportions of pregnancies with prescribed medication pairs – sensitivity analysis.

**Additional file 8: Figure S4.** Risk factors associated with polypharmacy during the first trimester of pregnancy.

**Additional file 9: Figure S5.** Risk factors associated with polypharmacy during the entire pregnancy period.

## Data Availability

The data that support the findings of this study are available from CPRD but restrictions apply to the availability of these data, which were used under license for the current study, and so are not publicly available.

## Abbreviations

BMI: Body mass index
BNF: British National Formulary
CPRD: Clinical Practice Research Datalink
FDA: US Food and Drug Administration
GP: General practitioner
NSAIDs: Non-steroidal anti-inflammatory drugs
SSRIs: Selective serotonin re-uptake inhibitors
UK: United Kingdom
