# Supplementary figures and images for: Polypharmacy during pregnancy and associated risk factors: a retrospective analysis of 577 medication exposures among 1.5 million pregnancies in the UK, 2000-2019
Source: BMC Med. 2023 Jan 16;21:21. doi: 10.1186/s12916-022-02722-5 (PMC9843951; doi:10.1186/s12916-022-02722-5)

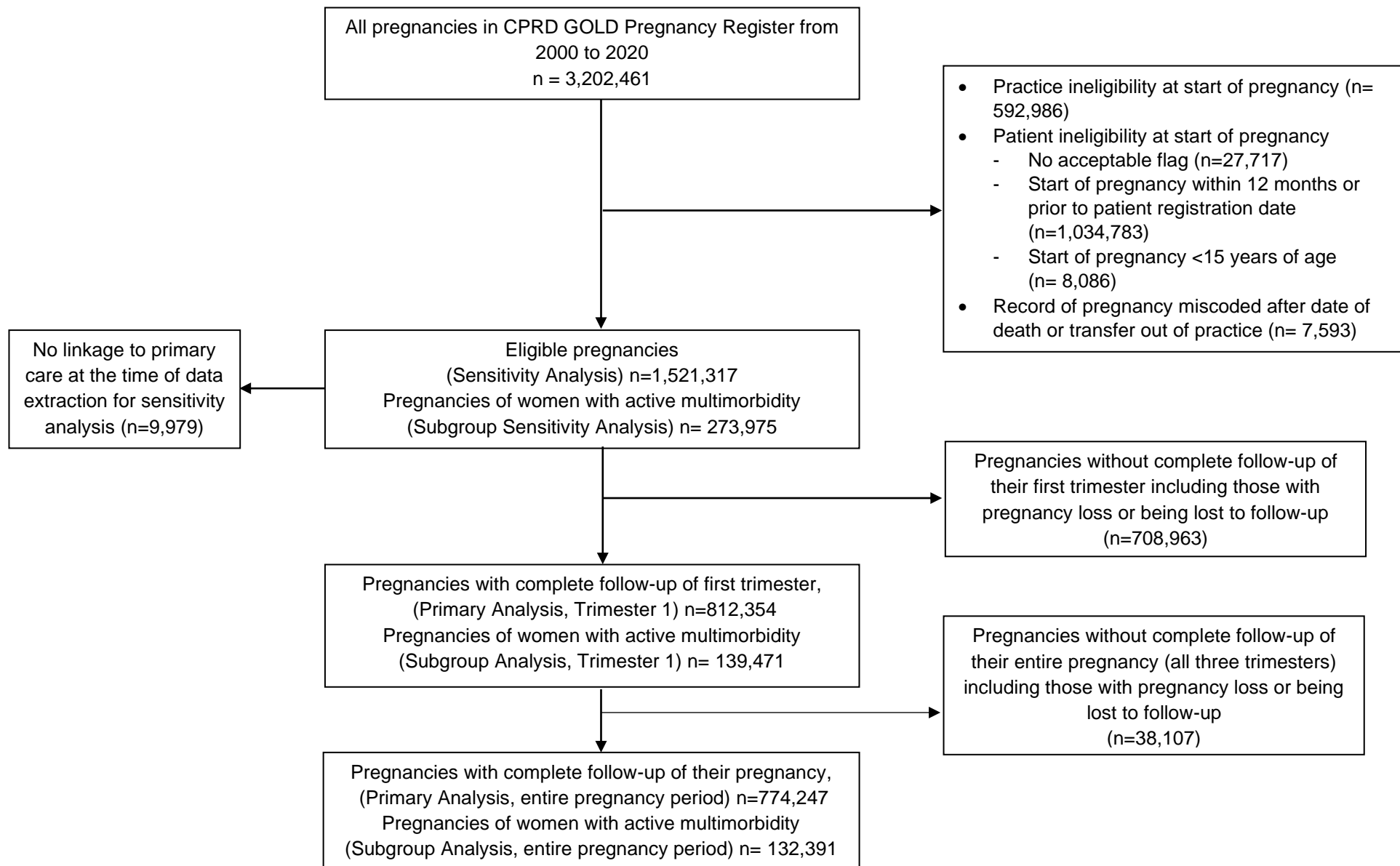

Supplement: Supplementary file 1 — Additional file 1: Figure S1. Flow diagram showing the selection of eligible pregnancies from the CPRD pregnancy register. [file 12916_2022_2722_MOESM1_ESM.pdf]

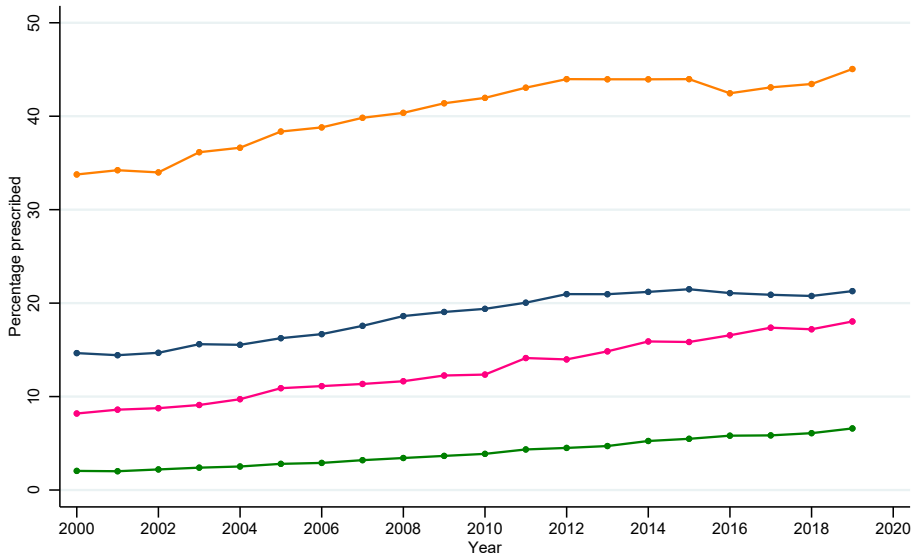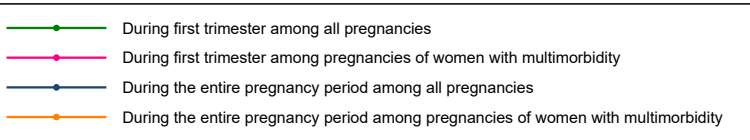

Supplement: Supplementary file 3 — Additional file 3: Figure S3. Polypharmacy (5 or more medications) prevalence trend from 2000 to 2019. [file 12916_2022_2722_MOESM3_ESM.pdf]

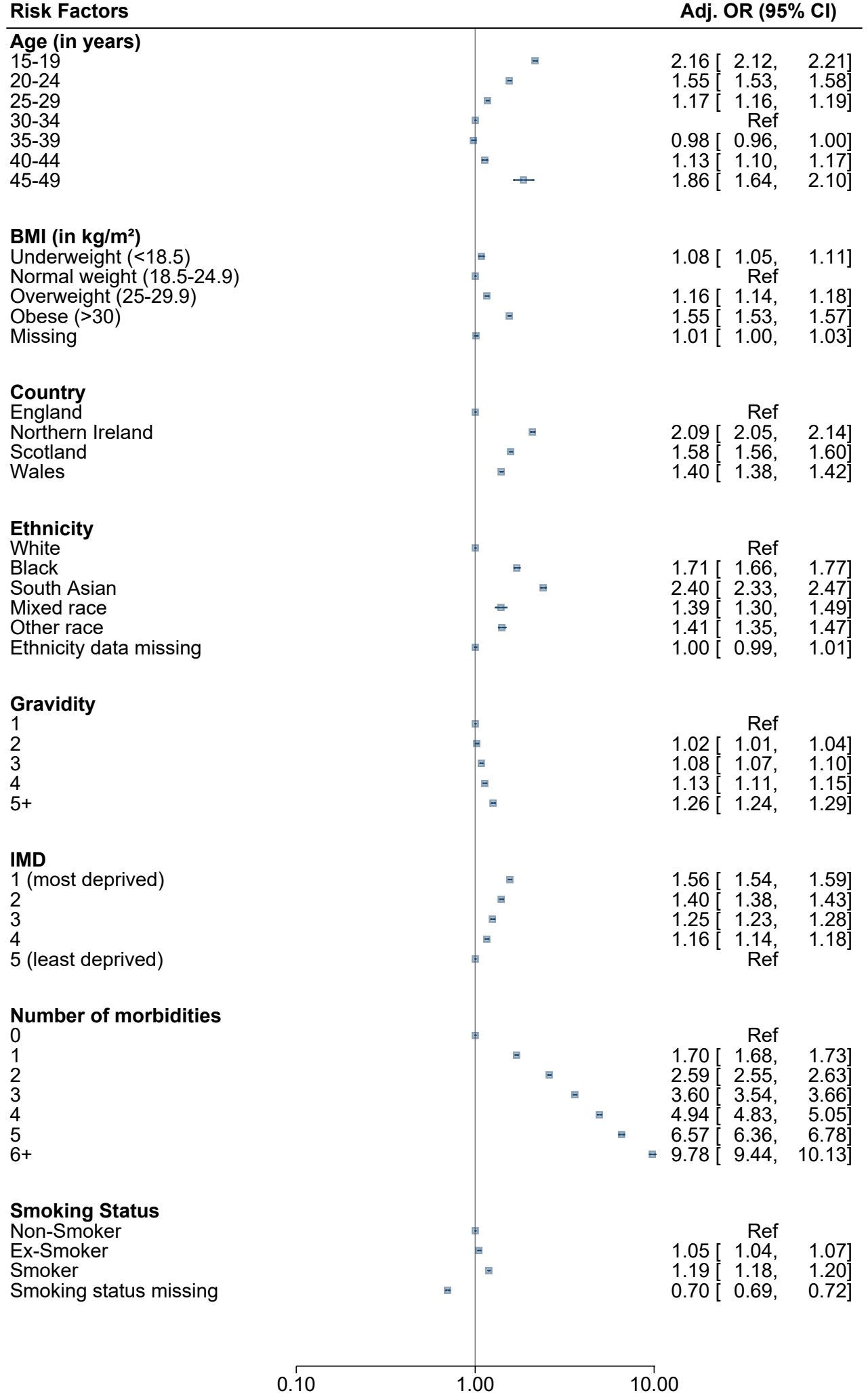

Supplement: Supplementary file 8 — Additional file 8: Figure S4. Risk factors associated with polypharmacy during the first trimester of pregnancy. [file 12916_2022_2722_MOESM8_ESM.pdf]

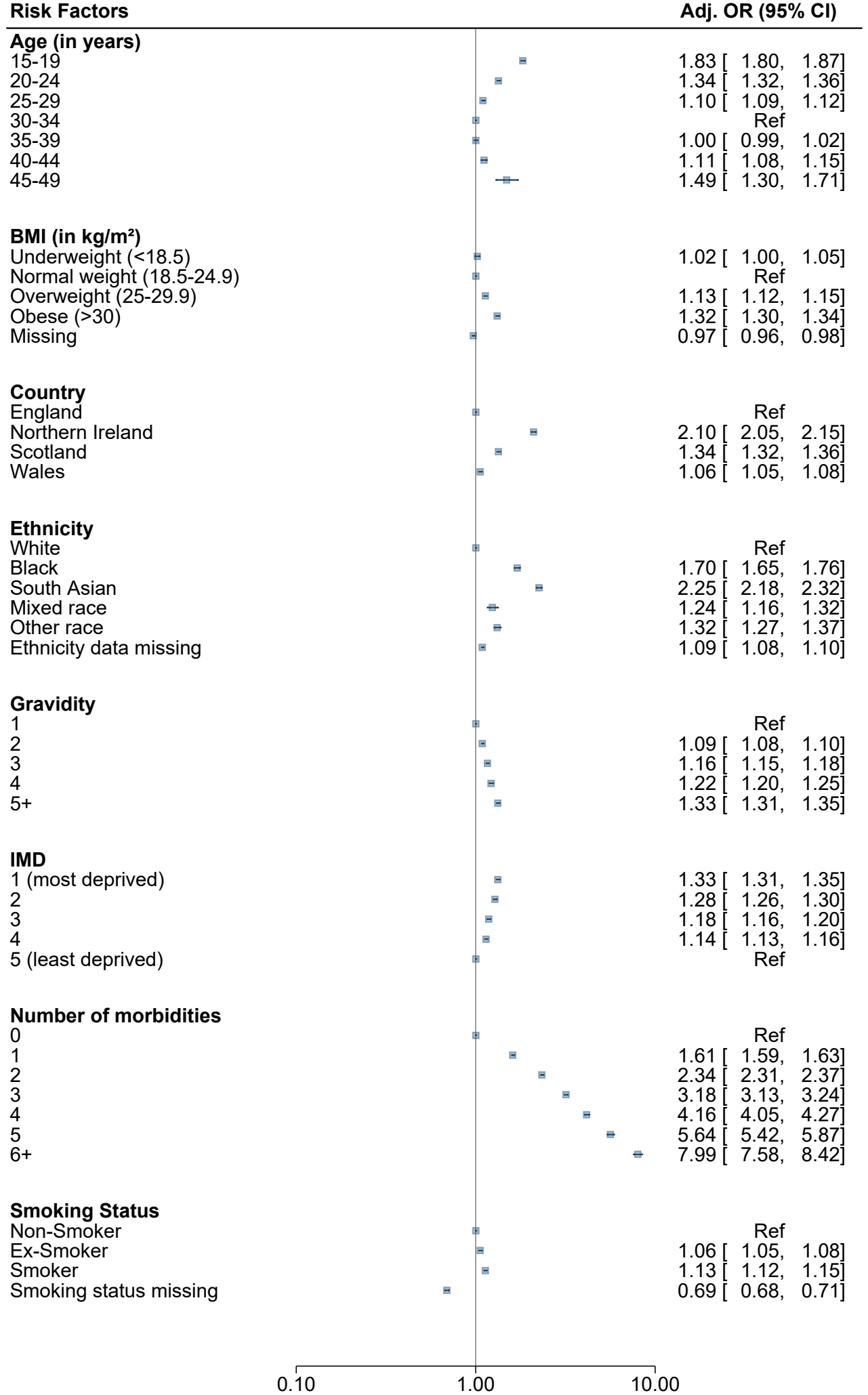

Supplement: Supplementary file 9 — Additional file 9: Figure S5. Risk factors associated with polypharmacy during the entire pregnancy period. [file 12916_2022_2722_MOESM9_ESM.pdf]
