# Supplementary material for: Polypharmacy during pregnancy and associated risk factors: a retrospective analysis of 577 medication exposures among 1.5 million pregnancies in the UK, 2000-2019
Source: BMC Med. 2023 Jan 16;21:21. doi: 10.1186/s12916-022-02722-5 (PMC9843951; doi:10.1186/s12916-022-02722-5)
Supplement: Supplementary file 2 — Additional file 2: Figure S2. Polypharmacy prevalence according to a range of definitions (2 to 11 or more medications) during (A) first trimester and (B) the entire pregnancy, among pregnancies of all eligible women and pregnancies of women with multimorbidity, with or without complete follow up (Sensitivity Analysis). [file 12916_2022_2722_MOESM2_ESM.pdf]

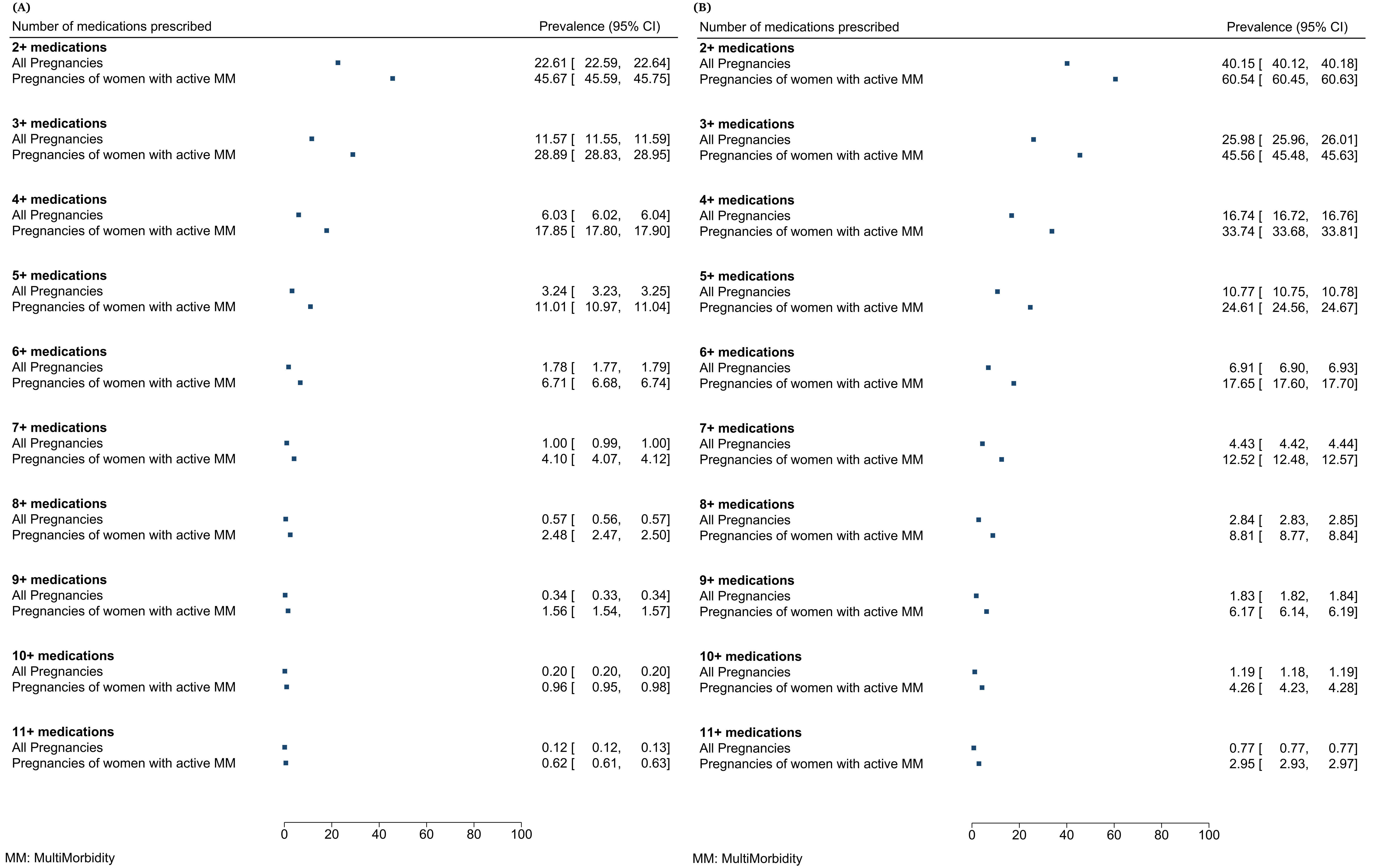
(A)
Number of medications prescribed
Prevalence (95% CI)
2+ medications
All Pregnancies 22.61 [22.59, 22.64]
Pregnancies of women with active MM 45.67 [45.59, 45.75]
3+ medications
All Pregnancies 11.57 [11.55, 11.59]
Pregnancies of women with active MM 28.89 [28.83, 28.95]
4+ medications
All Pregnancies 6.03 [6.02, 6.04]
Pregnancies of women with active MM 17.85 [17.80, 17.90]
5+ medications
All Pregnancies 3.24 [3.23, 3.25]
Pregnancies of women with active MM 11.01 [10.97, 11.04]
6+ medications
All Pregnancies 1.78 [1.77, 1.79]
Pregnancies of women with active MM 6.71 [6.68, 6.74]
7+ medications
All Pregnancies 1.00 [0.99, 1.00]
Pregnancies of women with active MM 4.10 [4.07, 4.12]
8+ medications
All Pregnancies 0.57 [0.56, 0.57]
Pregnancies of women with active MM 2.48 [2.47, 2.50]
9+ medications
All Pregnancies 0.34 [0.33, 0.34]
Pregnancies of women with active MM 1.56 [1.54, 1.57]
10+ medications
All Pregnancies 0.20 [0.20, 0.20]
Pregnancies of women with active MM 0.96 [0.95, 0.98]
11+ medications
All Pregnancies 0.12 [0.12, 0.13]
Pregnancies of women with active MM 0.62 [0.61, 0.63]
0 20 40 60 80 100
MM: MultiMorbidity
(B)
Number of medications prescribed
Prevalence (95% CI)
2+ medications
All Pregnancies 40.15 [40.12, 40.18]
Pregnancies of women with active MM 60.54 [60.45, 60.63]
3+ medications
All Pregnancies 25.98 [25.96, 26.01]
Pregnancies of women with active MM 45.56 [45.48, 45.63]
4+ medications
All Pregnancies 16.74 [16.72, 16.76]
Pregnancies of women with active MM 33.74 [33.68, 33.81]
5+ medications
All Pregnancies 10.77 [10.75, 10.78]
Pregnancies of women with active MM 24.61 [24.56, 24.67]
6+ medications
All Pregnancies 6.91 [6.90, 6.93]
Pregnancies of women with active MM 17.65 [17.60, 17.70]
7+ medications
All Pregnancies 4.43 [4.42, 4.44]
Pregnancies of women with active MM 12.52 [12.48, 12.57]
8+ medications
All Pregnancies 2.84 [2.83, 2.85]
Pregnancies of women with active MM 8.81 [8.77, 8.84]
9+ medications
All Pregnancies 1.83 [1.82, 1.84]
Pregnancies of women with active MM 6.17 [6.14, 6.19]
10+ medications
All Pregnancies 1.19 [1.18, 1.19]
Pregnancies of women with active MM 4.26 [4.23, 4.28]
11+ medications
All Pregnancies 0.77 [0.77, 0.77]
Pregnancies of women with active MM 2.95 [2.93, 2.97]
0 20 40 60 80 100
MM: MultiMorbidity
